# Selective Pharmacological Targeting of a DEAD Box RNA Helicase

**DOI:** 10.1371/journal.pone.0001583

**Published:** 2008-02-13

**Authors:** Lisa Lindqvist, Monika Oberer, Mikhail Reibarkh, Regina Cencic, Marie-Eve Bordeleau, Emily Vogt, Assen Marintchev, Junichi Tanaka, Francois Fagotto, Michael Altmann, Gerhard Wagner, Jerry Pelletier

**Affiliations:** 1 Department of Biochemistry, McGill University, Montreal, Quebec, Canada; 2 Department of Biological Chemistry and Molecular Pharmacology, Harvard Medical School, Boston, Massachusetts, United States of America; 3 Department of Biology, McGill University, Montreal, Quebec, Canada; 4 Department of Chemistry, Biology, and Marine Sciences, University of the Ryukyus, Nishihara, Okinawa, Japan; 5 Institut für Biochemie und Molekulare Medizin, Universität Bern, Bern, Switzerland; 6 McGill Cancer Center, McGill University, Montreal, Quebec, Canada; Victor Chang Cardiac Research Institute, Australia

## Abstract

RNA helicases represent a large family of proteins implicated in many biological processes including ribosome biogenesis, splicing, translation and mRNA degradation. However, these proteins have little substrate specificity, making inhibition of selected helicases a challenging problem. The prototypical DEAD box RNA helicase, eIF4A, works in conjunction with other translation factors to prepare mRNA templates for ribosome recruitment during translation initiation. Herein, we provide insight into the selectivity of a small molecule inhibitor of eIF4A, hippuristanol. This coral-derived natural product binds to amino acids adjacent to, and overlapping with, two conserved motifs present in the carboxy-terminal domain of eIF4A. Mutagenesis of amino acids within this region allowed us to alter the hippuristanol-sensitivity of eIF4A and undertake structure/function studies. Our results provide an understanding into how selective targeting of RNA helicases for pharmacological intervention can be achieved.

## Introduction

Helicases and translocases are classified into 6 superfamilies (SF1–SF6) based on the arrangement of conserved sequence motifs, with many providing essential functions in nucleic acid metabolic processes [1]. Members of the SF2 family consist of RNA helicases implicated in transcription, RNA export, splicing, translation, ribosome biogenesis, miRNA processing, and RNA decay [2]–[4]. Eukaryotic initiation factor (eIF) 4A is one of the archetypical founding members of the DEAD box helicase family, the largest subclass of the SF2 family. eIF4A is an abundant translation factor that exists in free form (referred to herein as eIF4A_f_) or as a subunit of the heterotrimeric cap binding complex, eIF4F (referred to herein as eIF4A_c_) [5], [6]. It participates in the ribosome recruitment phase of translation and is delivered to the cap structure (m7GpppN, where N is any nucleotide) of mRNA templates as a subunit of eIF4F. It is thought to prepare the mRNA template for 43S pre-initiation complex (40S ribosome and associated factors) binding by unwinding local secondary structure. The helicase activity of eIF4A_c_ is ∼20-fold more efficient than eIF4A_f_ [7], [8] and during initiation eIF4A_f_ is thought to cycle through the eIF4F complex [9]–[12]. There are two highly related isoforms, eIF4AI and eIF4AII (85–90% sequence identity) which are thought to be functionally interchangeable for translation initiation [12], [13]. A third protein, called eIF4AIII (DDX48), has ∼65% sequence identity to eIF4AI and is part of the exon junction complex that participates in nonsense mediated decay [14], [15]. The helicase activity of eIF4A is inhibited when associated with the tumor suppressor gene product, Pdcd4, an event that is regulated by the mammalian target of rapamycin (mTOR) [16], [17]. This underscores an important link between cellular homeostasis and translational control at the level of eIF4A availability.

In a screen aimed at identifying novel inhibitors of translation initiation, we identified and characterized two marine-derived natural products, pateamine and hippuristanol, that modulate eIF4A activity [18]–[20]. The binding site of pateamine on eIF4A is not defined, although its activity is dependent on the nature of the linker region joining the amino-terminal (NTD) and carboxy-terminal domains (CTD), a region with significant sequence variation among DEAD-box family members [21]. On the other hand, hippuristanol interacts with eIF4AI-CTD (residues 237–406) and blocks the RNA-dependent ATPase, RNA binding, and helicase activities of eIF4AI [20]. Herein, we define the hippuristanol-binding site on eIF4A. The site displays extensive sequence variation among DEAD box RNA helicases and provides a framework for understanding the selectivity of hippuristanol. We utilize this information to generate eIF4A alleles with reduced sensitivity to this small molecule and capable of rescuing hippuristanol-induced inhibition of translation. This allowed us to probe structure-function relationships of eIF4A in translation.

## Results

### Defining the eIF4A hippuristanol binding site

**Figure 1:**
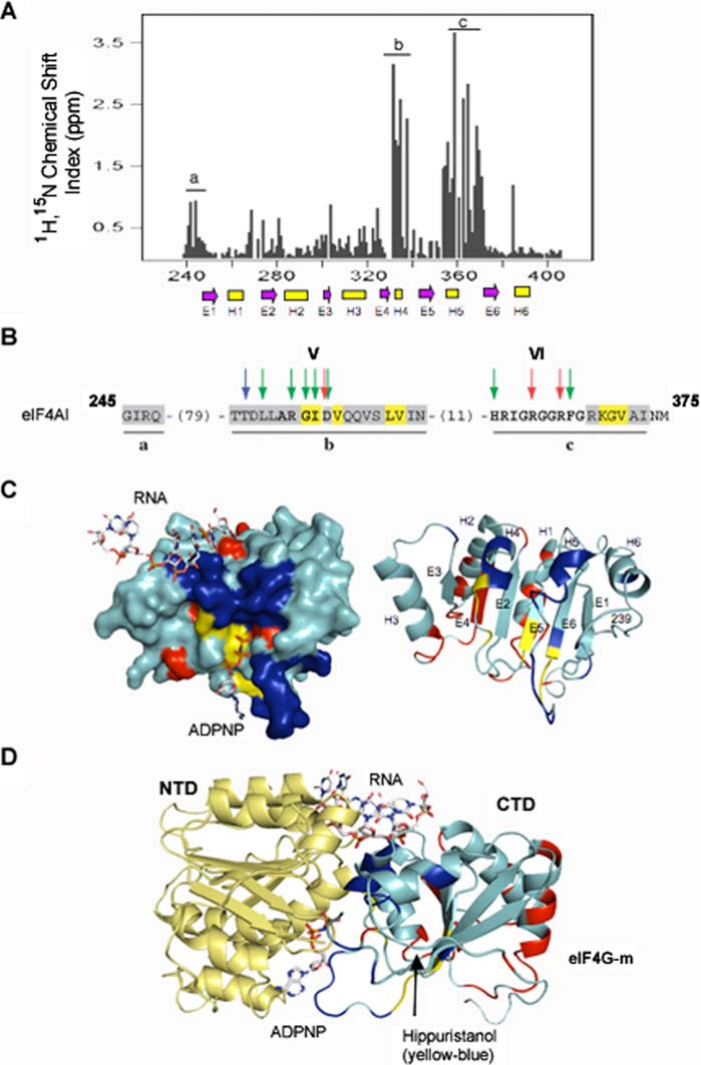
Hippuristanol binds to eIF4AI-CTD. (A) Chemical shift changes of ^1^H-^15^N-HSQC peaks, (Δδ(^1^H)+0.2 Δδ(^15^N), of eIF4A-I-CTD (52 µM) upon addition of hippuristanol (100 µM). Free and bound forms are in slow exchange and the resonances of eIF4AI-CTD had to be assigned in both states. The locations of secondary structures were identified by NMR and are indicated with magenta arrows (β-strands) and yellow rectangles (helices). (B) Primary amino acid sequence of eIF4AI indicating residues involved in hippuristanol binding. NOEs are highlighted in yellow, whereas those within 5Å are in grey and correspond to regions a, b, and c in A. Residues in bold denote conserved amino acids that define motifs V (ARGID) and VI (HRIGRGGRFG) of DEAD box family members [40]. Arrows denote residues identified in Vasa that interact with ATP (red), RNA (blue), or are involved in interdomain interaction (green)[25]. (C) Surface and ribbon representations of the model for eIF4AI-CTD. The CTD is viewed from the position of the NTD. Residues of eIF4AI-CTD that show NOEs to hippuristanol are coloured yellow, those exhibiting major chemical shift changes but no NOEs are coloured blue. Residues contacting eIF4G are in red [24]. The β-sheets (E1–E6) and α-helices (H1–H6) are labelled and refer to the locations marked in A. RNA and ADPNP are shown as sticks models. (D) Location of the hippuristanol-binding site in a model for eIF4AI complexed with RNA and ADPNP. The model is composed of the crystal structure of human eIF4AI-NTD (PDB #2G9N) and the homology model of the eIF4AI-CTD [24]. The two domains are aligned to the structure of eIF4AIII from the EJC from which the RNA and ADPNP binding sites are adapted (PDB# 2HYI) [22]. Color scheme of amino acid residues is as in C.

To identify the amino acids involved in hippuristanol binding, a series of NMR experiments were undertaken in which ^1^H-^15^N-HSQC spectra of uniformly labelled eIF4AI-CTD were obtained in the absence or presence of compound (Fig. 1A). Residues that experienced significant chemical shift changes (>mean plus standard deviation) are indicated in grey whereas those displaying direct NOE contacts (<5Å) are highlighted in yellow (Fig. 1B). Hippuristanol binds directly (exhibits NOEs) to the N-terminal ends of β-strands E5 and E6, the C-terminal end of helix H4, as well as the loop regions adjacent to these secondary structural elements (Figs. 1A–C). Furthermore, adjacent regions undergo significant chemical shift changes (Fig. 1C; highlighted in blue).

We analyzed the position of the hippuristanol binding site in the context of a model based on the domain orientation of eIF4AIII in the exon junction complex (EJC) (PDB code 2HYI) [22], [23] (Fig. 1D). Here, the eIF4AI NTD crystal structure (PDB #2G9N) was used and the eIF4AIII-CTD was replaced with the homology model for eIF4AI [24]. The positions of the RNA and ADPNP are taken from the EJC [22]. Accordingly, the hippuristanol-binding site on the eIF4AI-CTD is directly adjacent to the ATP-binding site in the NTD. Since hippuristanol does not inhibit ATP crosslinking to eIF4A [20], it may perturb the interface between the NTD and CTD domains. The hippuristanol-binding site is far from the RNA-binding face and allows us to conclude that hippuristanol inhibits eIF4A RNA binding in an allosteric manner.

### Selectivity of hippuristanol for eIF4A

With the exception of R247^eIF4AI^ and T328^eIF4AI/II^, all the hippuristanol binding residues are present in murine eIF4AI, eIF4AII, and the yeast eIF4A homolog Tif1/2p (Fig. S1). This hippuristanol binding site however is not conserved in eIF4AIII and we note 7 amino acid differences (Fig. S1; R247^eIF4AI^ is changed to K252^eIF4AIII^, T328^eIF4AI^ to S333^eIF4AIII^, L331^eIF4AI^ to V336^eIF4AIII^, L332^eIF4AI^ to W337^eIF4AIII^, I336^eIF4AI^ to L341^eIF4AIII^, Q339^eIF4AI^ to P344^eIF4AIII^, and V344^eIF4AI^ to I349^eIF4AIII^). Among the residues that differ between eIF4AI and eIF4AIII, the L341^eIF4AIII ^, P344^eIF4AIII^ and I349^eIF4AIII^ changes are expected to directly impact on hippuristanol binding since the corresponding side chains are part of the binding pocket (Fig. S1). The side chains of the other substitutions do not point towards the binding site and are not expected to impact on hippuristanol affinity.

**Figure 2:**
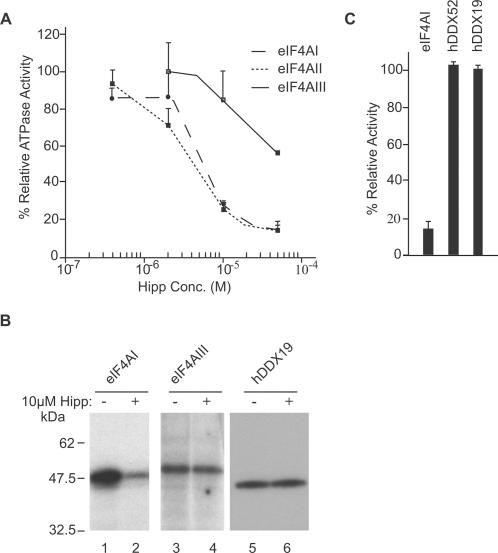
Selectivity of hippuristanol for eIF4A. (A) Inhibition of eIF4A RNA-dependent ATPase activity by hippuristanol. ATPase assays were performed with 0.1 µg of His_6_-eIF4AI or His_6_-eIF4AII at 25°C or with 0.1 µg of His_6_-eIF4AIII at 37°C for 2 h with 0.1 µCi γ-^32^P-ATP (10 Ci/mmol). Following analysis by TLC and quantitation using a Fuji BAS 2000 phosphoimager, the percent hydrolysis was determined and set relative to the DMSO vehicle control reactions. Each value represents the average of three measurements with the error shown as the standard deviation. (B) Crosslinking of recombinant proteins to RNA in the presence of hippuristanol. ^32^P-labelled CAT RNA was cross-linked to 0.5–1 µg of the indicated recombinant protein in the presence or absence of hippuristanol, separated by SDS-PAGE, and visualized by autoradiography. [Note that in our hands, recombinant hDDX52 did not crosslink to RNA.] (C) Relative ATPase activity of eIF4AI, hDDX19, and hDDX52 in the presence of 50 µM hippuristanol. eIF4AI and hDDX19 where performed at 25°C for 5 minutes while hDDX52 was incubated for 60 minutes to allow for analysis to be in the linear range of ATP hydrolysis. The percent ATP hydrolysis was determined in the presence of hippuristanol and set relative to the DMSO vehicle control reactions. The results represent the average of 3 experiments with error bars signifying the standard deviation.

We compared the relative sensitivities of murine eIF4AI, murine eIF4AII, and human eIF4AIII to hippuristanol in an RNA-dependent ATPase assay (Fig. 2A). eIF4AI and eIF4AII showed similar sensitivities to hippuristanol, whereas eIF4AIII required ∼10-fold higher concentrations of compound to achieve equivalent inhibition (Fig. 2A). Tif1/2p ATPase activity displayed a similar sensitivity to hippuristanol as eIF4AI/II (data not shown), consistent with the conserved nature of the amino acids flanking motifs V and VI (Fig. S1). Consistent with these results, 10 µM hippuristanol inhibited RNA binding of eIF4AI, but not eIF4AIII (Fig. 2B, compare lane 2 to 1 and lane 4 to 3).

Alignment of the region encompassing the hippuristanol binding site of murine (Fig. S2A) and human (Fig. S2B) DEAD box helicases indicates extensive sequence variation. Human DDX52 has a very high degree of conservation with eIF4AI in the hippuristanol binding region, with two changes present among the amino acids showing direct NOE contacts (Fig. S2B). Eleven of 17 amino acids (from regions encompassing motifs V and VI) showing weaker NOEs are also present in hDDX52 (Fig. S2B). hDDX19 contains five of the eight amino acids showing direct NOE contacts and 11 of the 17 amino acids showing weaker NOEs (Fig. S2B). Hippuristanol did not inhibit the RNA binding properties of hDDX19 (Fig. 2B) nor the ATPase activity of hDDX52 or hDDX19 (Fig. 2C). These results provide insight into why hippuristanol is selective for eIF4A since the amino acids that define the hippuristanol binding site are not well conserved among other DDX family members.

### Modulating eIF4A Hippuristanol Sensitivity

Using the mapping information from these NMR studies, we addressed the feasibility of modulating hippuristanol sensitivity among eIF4A family members (Fig. S1). Given that Ded1p is resistant to inhibition by hippuristanol (Hipp^R^), we used information obtained from the sequence comparison of the hippuristanol binding site to guide us in our mutagenesis approach [20] (Fig. S1). The ^338^VQ^339^ eIF4AI amino acid pair immediately downstream of motif V in eIF4A was altered to ^338^IP^339^ (present in Ded1p) or ^338^IG^339^ (for future NMR studies; since proline residues do not have amide protons and are not visible in ^1^H-^15^N HSQC spectra). These mutants also harboured a G363T alteration in motif VI (eIF4AI^IG/T^), so we generated mutants harbouring only either a G363T or a ^338^VQ^339 ^to ^338^IG^339^ alteration (Fig. S1; eIF4AI^IG^ and eIF4AI^T^). IP/T variants of eIF4AII and eIF4AIII were also generated (Fig. S1). We also addressed whether we could increase the sensitivity of eIF4AIII to hippuristanol by rebuilding a complete hippursitanol site (Fig. S1; eIF4AIII^TLLQV^).

**Figure 3:**
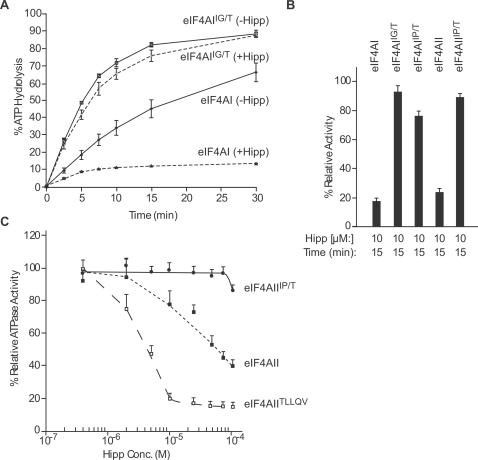
Characterization of eIF4A hippuristanol-resistant mutants. (A) Consequence of mutations in the eIF4AI hippuristanol binding site on ATP hydrolysis. ATP hydrolysis was monitored using 1 µg His_6_-eIF4AI or His_6_-eIF4AI^IG/T^ in the presence or absence of 10 µM hippuristanol. Each value represents the average of three measurements with the standard deviation presented. (B) Relative ATPase activity of eIF4A mutants in the presence of hippuristanol. The percent ATP hydrolysis was determined in the presence of hippuristanol and set relative to the values obtained in the presence of control reactions containing vehicle (DMSO). The results represent the average of 3–7 experiments with error bars signifying the standard deviation. (C) Altered hippuristanol sensitivity of eIF4AIII. ATPase assays were performed with 0.5 µg recombinant protein with 0.1 µCi γ-^32^P-ATP (10 Ci/mmol). Following analysis by TLC and quantitation using a Fuji BAS 2000 phosphoimager, the percent hydrolysis was determined and set relative to the DMSO vehicle control reactions. Each value represents the average of three measurements with the standard deviation shown.

eIF4AI^IG/T^ and eIF4AI^IP/T^ are more active than eIF4AI in an RNA-dependent ATPase assay (Fig. 3A and data not shown). The increased ATPase activity was principally a consequence of the ^338^VQ^339^ to ^338^IG^339 ^alteration (Fig. S3). Hippuristanol inhibited eIF4AI ATPase activity but had little effect on the ATPase activity of eIF4AI^IG/T^ and eIF4AI^IP/T^ (Fig. 3A and B). Similarly, eIF4AII was sensitive to inhibition by hippuristanol whereas eIF4AII^IP/T^ was not (Fig. 3B). Titration of hippuristanol revealed that the ATPase activity of eIF4AIII^IP/T ^was resistant to hippuristanol at concentrations up to 75 µM with a slight inhibition of activity at 100 µM (Fig. 3C). In contrast, eIF4AIII^TLLQV^ was more sensitive to hippuristanol than eIF4AIII (Fig. 3C), showing a dose-response profile that resembled that of eIF4AI and eIF4AII (Fig. 2A). We further characterized the eIF4AI and eIF4AII hippuristanol-resistant alleles in RNA binding and helicase assays. As expected, hippuristanol reduced the ability of eIF4AI and eIF4AII to interact with RNA (Fig. S4A, compare lanes 2 and 8 to 1 and 7, respectively) but did not affect the RNA binding activities of eIF4AI^IG/T^, eIF4AI^IP/T^, and eIF4AII^IP/T^ (Fig. S4A, compare lanes 4, 6, and 10 to 3, 5, and 9, respectively). The helicase activity of eIF4AI and eIF4AII is blocked by hippuristanol, whereas both eIF4AI^IG/T ^and eIF4AII^IP/T ^are resistant to inhibition (Fig. S4B). Taken together, these results demonstrate the feasibility of generating mutant alleles of DEAD-box helicase members with increased or reduced sensitivity to hippuristanol. This provides a powerful means by which to investigate the function of individual members of this family of proteins.

### Structure/Function Studies of eIF4A

We used the ability to generate hippuristanol-resistant alleles of eIF4A to probe structure-function relationships *in vitro*. Specifically, we asked: (i) if the helicase activity of eIF4AI is required for translation (or is its ATPase activity sufficient); (ii) if eIF4A:eIF4G interaction is essential for translation, and (iii) whether eIF4AI and eIF4AII are functionally interchangeable. The design of a helicase deficient mutant of eIF4A, eIF4AI^Hel/IG/T^, was guided by a previously described Vasa mutation in which this alteration abolished helicase activity but only reduced ATPase activity by 50% (Fig. S1) [25]. eIF4AI^Quad/IG/T^ contains 4 missense mutations previously shown to inhibit interaction with eIF4G [24]. eIF4AI^Hel/IG/T^ showed a reduction in the rate of RNA-dependent ATP hydrolysis compared to eIF4AI^IG/T^ - bringing it to levels similar to wild-type eIF4AI (Fig. S5A). As expected, eIF4AI^Hel/IG/T^ does not display helicase activity (Fig. S5B, compare lanes 5 to 3), and its RNA binding activity is resistant to hippuristanol (Fig. S5C). eIF4AI^Quad/IG/T^ has a similar rate of RNA-dependent ATP hydrolysis as eIF4AI^IG/T^ (Fig. S5A), possesses helicase activity (Fig. S5D), and its RNA binding activity is resistant to hippuristanol (Fig. S5C). As predicted, it is impaired in its ability to interact with eIF4GI (Fig. S6).

**Figure 4:**
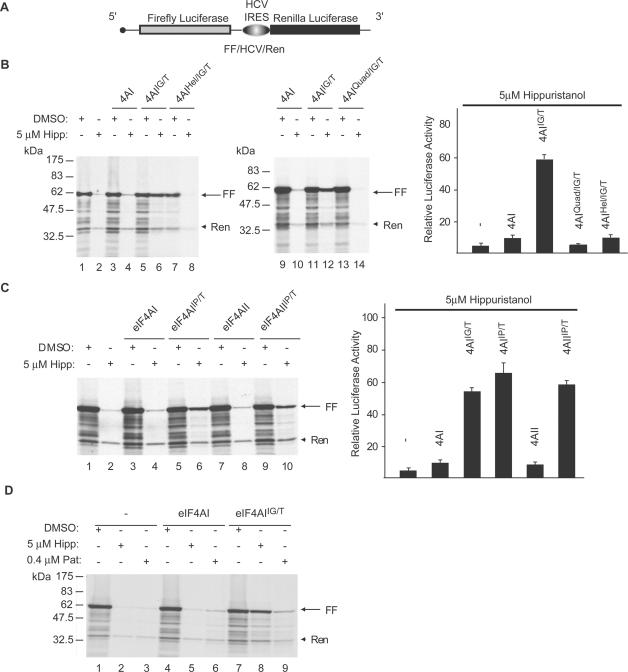
Functional requirements for eIF4A activity in translation. (A) Schematic representation of the reporter construct used in these studies is shown on top. (B) Left Panel: Rescue of hippuristanol-induced translation inhibition by eIF4AI^IG/T^. *In vitro* translations in RRL programmed with capped FF/HCV/Ren mRNA (8 µg/ml) and containing vehicle (0.1% DMSO) or 5 µM hippuristanol (in 0.1% DMSO) were supplemented with 0.5 µg recombinant protein. Protein synthesis was assessed by using ^35^S-methionine incorporation as well as by monitoring luciferase assays. Protein products were separated by SDS-PAGE and visualized by autoradiography. The arrow indicates the position of migration of the firefly luciferase, whereas the arrowhead denotes the position of migration of *Renilla* luciferase. Right panel: Relative luciferase activity obtained in the presence of recombinant eIF4A. *Firefly* RLU readings obtained in the presence of recombinant eIF4A and hippuristanol were standardized to *Renilla* RLU values and set relative to the values obtained in the presence of vehicle (DMSO). The average of 3–8 experiments is shown with the standard deviations denoted. (C) eIF4AI and eIF4AII are functionally interchangeable. *In vitro* translations in RRL containing vehicle (DMSO) or 5 µM hippuristanol were supplemented with 0.8 µg recombinant eIF4A where indicated, and programmed with capped FF/HCV/Ren mRNA (8 µg/ml). Left panel: Protein products were separated by SDS-PAGE and visualized by autoradiography. The arrow indicates the position of migration of the firefly luciferase, whereas the arrowhead denotes the position of migration of *Renilla* luciferase. Right panel: Relative luciferase activity obtained in the presence of recombinant eIF4A and hippuristanol was standardized to *Renilla* Luciferase levels and set relative to the values obtained in the presence of vehicle (DMSO). The average of 4–5 experiments is shown with the standard deviations denoted. (D) Translational rescue by eIF4AI^IG/T ^is selective for hippuristanol. *In vitro* translations in RRL containing vehicle (DMSO), 5 µM hippuristanaol, or 0.4 µM pateamine were supplemented with 0.8 µg recombinant eIF4A where indicated, and programmed with capped FF/HCV/Ren mRNA (8 µg/ml). Protein products were separated by SDS-PAGE and visualized by autoradiography. The arrow indicates the position of migration of the firefly luciferase, whereas the arrowhead denotes the position of migration of *Renilla* luciferase. The figure is a representative display of one of two experiments.

We tested whether the Hipp^R^ eIF4A alleles could rescue translation when this process is inhibited with hippuristanol (Fig. 4). *In vitro* translations were performed in rabbit reticulocyte lysate (RRL) programmed with the bicistronic reporter mRNA FF/HCV/Ren (Fig. 4A) [20]. Here, *Renilla* (Ren) luciferase expression is HCV-driven and not eIF4A-dependent [20], thus serving as an internal control. Firefly (FF) luciferase expression is inhibited by >90% in the presence of 5 µM hippuristanol, whereas that of *Renilla* is slightly reduced (Fig. 4B, compare lane 2 to 1). *Renilla* luciferase RLU readings from the translation products of this experiment are consistent with a 2-fold reduction in activity (LL, data not shown). Addition of wild-type eIF4AI does not rescue the inhibition by hippuristanol, whereas eIF4AI^IG/T ^restored translation to ∼60% of normal levels (Fig. 4B, compare lanes 6 and 4 to 5 and 3, respectively). These results are consistent with the idea that inhibition of translation by hippuristanol *in vitro* is a direct consequence of impaired eIF4A activity. Neither eIF4AI^Quad/IG/T^ or eIF4AI^Hel/IG/T^ are able to rescue translation inhibition by hippuristanol (Fig. 4B) indicating that eIF4A’s helicase activity and its ability to interact with eIF4G are necessary for its role in translation.

Next, we tested if eIF4AI and eIF4AII are functionally redundant for translation. To this end, we assessed the ability of eIF4AI^IP/T ^and eIF4AII^IP/T ^to rescue hippuristanol-induced translation inhibition (Fig 4C). Like eIF4AI^IG/T ^(Fig. 4B), both eIF4AI^IP/T ^and eIF4AII^IP/T ^rescued to the same extent (Fig. 4C, compare lanes 10 and 6 to 8 and 4, respectively). The rescue by the Hipp^R^ mutants in these experiments was specific for hippuristanol, since it was not observed when translation was inhibited by pateamine - another eIF4A small molecule activity modulator (Fig. 4D, compare lane 9 to 8).

### Hippuristanol targets eIF4A *in vivo*

**Figure 5:**
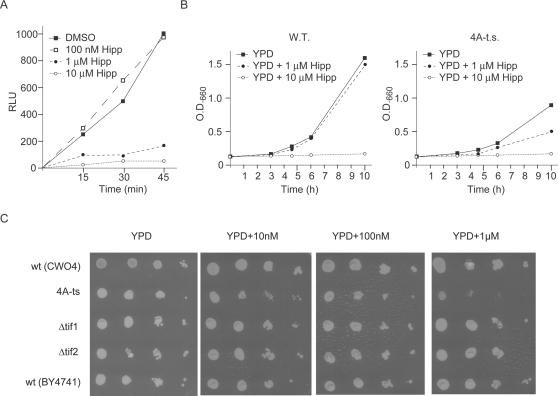
Hippuristanol targets eIF4A *in vivo*. (A) Inhibition of *Renilla* luciferase reporter in a yeast *in vitro* translation extract. Hippuristanol was added to a *S. cerevisiae* cytosolic translation extract programmed with 0.12 µg/ml capped *Renilla* luciferase mRNA. At various points following initiation of the translation reaction, aliquots were removed and the relative luciferase units (RLU) determined. (B) Haploinsufficiency for Tif1/2p leads to increased sensitivity to hippuristanol *in vivo.* Haploid wild type cells (strain CWO4) or an isogenic strain carrying the temperature-sensitive tif1V79A allele (strain SS13-3A/pSSC120) [41] were cultivated in rich medium (YPD) at 27°C to an O.D._600_ of 0.2, at which point hippuristanol (1 µM or 10 µM final concentration) or solvent (DMSO, 0.1%) was added and the growth of different cultures was monitored for several hours by measuring the O.D._600_. (C) Serial dilutions of different haploid yeast strains were plated on YPD-plates containing the indicated concentrations of hippuristanol and incubated for 2–3 days at 27°C: wt (CWO4), wild type strain CWO4; 4A-ts, strain SS13-3A/pSSC120 carrying the tif1V79A allele; Δtif1, a BY4741-derivative strain carrying a tif1::kanX deletion; Δtif2, a BY4741-derivative strain carrying a tif2::kanX deletion; wt (BY4741), wild type strain BY4741.

We used a genetic approach to demonstrate that hippuristanol targets eIF4A *in vivo*. *S. cerevisiae* contains two eIF4A orthologues of identical amino acid sequence, called Tif1 and Tif2 [26]. We first assessed whether hippuristanol can block translation in an *in vitro S. cerevisiae* system programmed with *Renilla* mRNA (Fig. 5A). Concentrations of 1 µM hippuristanol were sufficient to inhibit protein synthesis (Fig. 5A). If Tif1/2p is the relevant biological target of hippuristanol *in vivo*, then *Saccharomyces cerevisiae* strains showing reduced activity of Tif1/2p should be more sensitive to growth inhibition by this compound than the wild-type (wt) strain [26]. The growth of 4A-ts, which contains the temperature sensitive V69S allele of Tif1p, was more sensitive than the wild-type parental strain or strains containing a deletion of Tif1p (Δtif1) or Tif2p (Δtif2) (Figs. 5B–C). These results imply that hippuristanol targets the yeast homologue of eIF4A (Tif1/2p) *in vivo* to affect growth.

**Figure 6:**
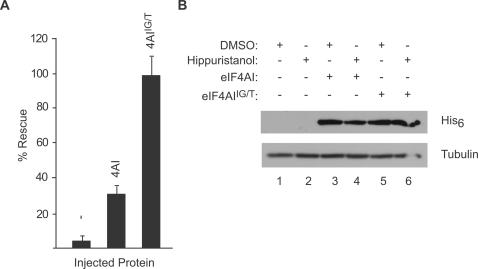
*In vivo* rescue of hippuristanol-induced translation inhibition by eIF4AI^IG/T^. (A) Rescue of translation in *Xenopus* oocytes by eIF4AI^IG/T^. The percent rescue was determined by normalizing the *Firefly* luciferase values to *Renilla* luciferase [to standardize for small variations in sample injection volumes], followed by dividing by the ratio obtained from the vehicle-treated samples (which was set at 100%). The data presented is the average of 9 independent sets of injections with the standard deviations denoted. (B) Western blot of extracts prepared from oocyte extracts. The equivalent of one oocyte was separated on a 10% SDS-PAGE, transferred to Immobilon-P, and probed with α-His_6_ (to detect recombinant His_6_-eIF4AI) or α-tubulin antibodies.

These findings prompted us to use the *Xenopus laevis* translation system to assess if hippuristanol-induced inhibition of translation could be relieved by eIF4AI^IG/T^ *in vivo* (Fig. 6). The hippuristanol binding site is 100% conserved between the murine and *X. laevis* eIF4AI proteins (data not shown). Addition of 5 µM hippuristanol inhibited cap-dependent translation of injected FF/HCV/Ren mRNA by 95% (Fig. 6A). Co-injection of recombinant eIF4AI slightly relieved the inhibition by hippuristanol to 30% of vehicle treated cells, whereas introduction of eIF4AI^IG/T ^completely rescued the effect (Fig. 6A). Equivalent amounts of recombinant eIF4AI and eIF4AI^IG/T ^were delivered to the cells upon micro-injection, as assessed by western blot analysis of extracts prepared from the injected eggs (Fig. 6B).

## Discussion

Herein, we demonstrate that hippuristanol interacts with amino acids within and adjacent to motifs V and VI of eIF4AI, two regions implicated in RNA, ATP, and interdomain contacts [23], [25]. Hippuristanol inhibits the RNA-dependent ATPase activity and RNA binding ability of eIF4A, but does not prevent binding of ATP to eIF4A [20]. The hippuristanol-binding site on eIF4A-CTD is adjacent to, or overlapping with, the NTD- and ATP-interacting surfaces. Since hippuristanol does not bind to the NTD, ATP would still be able to bind to eIF4A-NTD (its main binding site) in the presence of hippuristanol. The amino acid corresponding to Thr^329^ of eIF4AI (Fig. S1) in Vasa and eIF4AIII is implicated in RNA binding via interaction with a phosphate residue on the RNA backbone. We therefore speculate that either: (i) hippuristanol interferes with proper interdomain interaction, which in turn abolishes RNA binding or (ii) affects alignment of Thr^329^ with its target phosphate on RNA. We favor the former possibility as this mechanism of action has been documented by Nakamura and colleagues, who identified an RNA aptamer that inhibits eIF4A activity by also interfering with interdomain interaction [27].

Our results extend previous studies implicating eIF4A’s helicase activity and eIF4A:eIF4G interaction as being essential for translation (Fig. 4). eIF4AI^IG/T^, but not eIF4AI^Quad/IG/T^, was capable of rescuing hippuristanol-induced translation inhibition (Fig. 4), implying that eIF4AI^IG/T^ can assemble into the eIF4F complex and does not rescue translation as a free subunit. Consistent with this interpretation, rescue of translation by eIF4AI^IG/T^ is inhibited by the cap analog (m^7^GDP) (data not shown). Although eIF4A plays an accessory role in promoting 48S complex formation on unstructured mRNA templates, it is required for 48S complex formation and translation of mRNAs containing weakly structured (−13.6 kcal/mol) hairpins [28]. eIF4A has been proposed to cycle through the eIF4F complex during initiation [12] and mutants of eIF4A have been previously described which appear to act in a dominant-negative manner to trap eIF4F in an inactive state, thus inhibiting translation [10], [11]. The inverse relationship between 5′ secondary structure and sensitivity to inhibition by a dominant-negative mutant of eIF4A [11] is consistent with the idea that eIF4A functions to unwind secondary structure in the 5′ UTR during initiation to create a ribosome landing pad. However, not all potential activities of DEAD box proteins are necessarily involved in their function. Case in point is eIF4AIII where ATP hydrolysis is not required for its participation in NMD [29]. Therefore, we directly tested the requirement for eIF4A’s helicase activity in translation using a hippuristanol-resistant helicase-defective mutant and found it to be essential for eIF4A’s participation in initiation (Fig. 4). We also find that eIF4AI and eIF4AII are interchangeable in their ability to support translation *in vitro*. Previous reports documented that both eIF4AI and eIF4AII can associate with the eIF4F complex [12], [13], and our studies extend these results by demonstrating that an eIF4A mutant lacking this function cannot participate in the translation process. The ability to generate hippuristanol-sensitive alleles of various DDX family members is a powerful tool with which to assign function and undertake structure-function studies.

## Materials and Methods

### NMR Spectroscopy and modeling

NMR spectra were recorded at 298 K on a Varian Inova 600 and Inova 500 instruments equipped with cryogenic probes. Samples for NMR measurements typically contained 0.4–1 mM protein in buffer containing 20 mM Tris-HCl_7.0_, 300 mM NaCl, 5 mM DTT, 1 mM EDTA, 0.01% NaN_3_, 0.2 mM AEBSF, and 10% D_2_O. The spectra were processed with NMRPipe [30] and analyzed with XEASY[31]. Sequential resonance assignments for eIF4AI-CTD and its complex with hippuristanol were obtained from standard triple-resonance NMR experiments [HNCA, HN(CA)CB, HN(CO)CA, HN(COCA)CB, HNCO, HN(CA)CO] on uniformly ^15^N-/^13^C-labeled samples with 70% deuteration. ^15^N-edited NOESY-HSQC and TOCSY-HSQC experiments were recorded on uniformly ^15^N-labeled samples. Intermolecular NOEs between eIF4AI-CTD and hippuristanol were measured using uniformly ^15^N/^13^C/^2^H labelled CTD (52 µM) complexed with 100 µM hippuristanol. ^15^N-dispersed NOESY spectra were recorded in H_2_O, and exhibited intermolecular NOESY cross peaks between peptide HN and hippuristanol in an otherwise empty spectral region as described previously [32].

Homology modeling of the eIF4AI-CTD was previously described [24]. The model of full-length eIF4AI was constructed by manually superimposing the structure of human eIF4A-NTD (PDB# 2G9N) and the homology model of eIF4AI-CTD with the structure of eIF4AIII from the exon-junction complex (PDB# 2HYI) [22].

### Recombinant DNA Constructs

Site-directed mutagenesis was performed using a PCR based strategy. Oligonucleotides harboring the mutant sequence(s) were used in amplification reactions with either upstream or downstream oligonucleotides spanning unique restriction sites (Table SI). Following gel purification of PCR products, these were combined and used in amplification reactions in the absence of primer for 5 cycles, at which point primers targeting the ends of the fragments were added and amplifications continued for 30 cycles. DNA fragments were cloned into expression plasmids using convenient restrictions sites and all clones used in this study were sequenced to ensure the absence of secondary undesired mutations. The murine eIF4AI cDNA was subcloned from pET3b/eIF4AI into pET15b using NdeI and BamHI restriction enzymes [10]. The murine eIF4AII was excised from pACT2/4AII with BamHI (which was rendered blunt by Klenow repair) and XhoI, and inserted into the BamHI (blunt ended with Klenow)/XhoI sites of pET28a. Human eIF4AIII mutants were derived from pET-HTG-eIF4AIII [29] and subcloned into pET28a (BamHI site).

Cloning strategies to generate the various mutants utilized in this study were as follows: For pET15b/eIF4AI^IG^, two PCR products were produced using pET15b/eIF4AI as template and the primer pairs: (i) DraIII Forward and IG Reverse and (ii) IG Forward and BamHI Reverse (Table S1). Following gel purification, the fragments were used to extend off of each during 5 rounds of PCR amplification, after which the primers DraIII Forward and BamHI Reverse were used to amplify the full length mutant. The product was digested with DraIII and BamHI and inserted into pET15b/eIF4AI. For pET15b/eIF4AI^T^, two PCR products were produced using pET15b/eIF4AI as template and the primer pairs: (i) DraIII Forward and T Reverse and (ii) T Forward and BamHI Reverse (Table S1). The products were combined and cloned as described for pET15b/eIF4AI^IG^. For pET15b/eIF4AI^IG/T^, two PCR products were produced using pET15b/eIF4AI^IG^ as template and the primer pairs: (i) DraIII Forward and T Reverse and (ii) T Forward and BamHI Reverse (Table S1). The products were combined and cloned as described for pET15b/eIF4AI^IG^. For pET15b/eIF4AI^IP/T^, two PCR products were produced using pET15b/eIF4AI as template and the primer pairs: (i) DraIII Forward and IP Reverse and (ii) IP Forward and BamHI Reverse (Table S1). The products were combined and cloned as described for pET15b/eIF4AI^IG.^ Plasmid pET15b/eIF4AI^IP^ was used as template for two PCR reactions, using either (i) primers DraIII Forward and T Reverse and (ii) T Forward and BamHI Reverse. The products were combined and cloned as described for pET15b/eIF4AI^IG ^to generate pET15b/eIF4AI^IP/T^.

For pET15b/eIF4AI^Quad/IG/T^, two PCR products were produced using pET15b/eIF4AI^IG/T ^as template and the primer pairs: (i) primers D265R/E268Kforward and 4A(1220–1238)(AS) and (ii) D265R/E268Kreverse and pET15b Oligo (Table S1). Following gel purification, the fragments were used to extend off of each during 5 rounds of PCR amplification, after which the primers pET15b Oligo and 4A(1220–1238)(AS) were used to amplify the full length mutant. The product was digested with NdeI and BamHI and inserted into the same sites in pET15b to create pET15b/eIF4AI^RK/IG/T^. An additional round of mutagenesis was undertaken using this as template and the primer pairs: (i) D296A/T298Kreverse and pET15b Oligo, and (ii) D296A/T298Kforward and 4A(1220–1238)(AS) (Table S1). Following gel purification, the fragments were used to extend off of each other during 5 rounds of PCR amplification, after which the primers pET15b Oligo and 4A(1220–1238)(AS) were used to amplify the full length mutant. The product was digested with NdeI and BamHI and inserted into the same sites in pET15b to create pET15b/eIF4AI^Quad/IG/T^. For pET15b/eIF4AI^Hel/IG/T^, two PCR products were produced using pET15b/eIF4AI^IG/T ^as template and the primer pairs: (i) AIGforward and 4A(1220–1238)(AS) and (ii) AIGreverse and pET15b Oligo (Table S1). Following gel purification, the fragments were used to extend off of each during 5 rounds of PCR amplification, after which the primers pET15b Oligo and 4A(1220–1238)(AS) were used to amplify the full length mutant. The product was digested with NdeI and BamHI and inserted into the same sites in pET15b.

For pET28a/eIF4AII^IP/T^, two PCR products were produced using pET28a/eIF4AII as template and the primer pairs: (i) 4AII-IP(S) and 4AII(1278–1258) and (ii) 4AII-NheI-NTD and 4AII-IP(AS) (Table S1). Following gel purification, the fragments were used to extend off of each during 5 rounds of PCR amplification, after which the primers 4AII(1278-1258) and 4AII-NheI-NTD were used to amplify the full length mutant. The product was digested with NheI and XhoI and inserted into the same sites in pET28a to generate pET28a/eIF4AII^IP^. The T mutation was introduced (Fig. S1) using pET28a/eIF4AII^IP^ as template and the primer pairs: (i) 4AII-T(S) and 4AII(1278–1258) and (ii) 4AII-NheI-NTD and 4AII-T(AS) (Table S1). Following gel purification, the fragments were used to extend off of each during 5 rounds of PCR amplification, after which the primers 4AII(1278–1258) and 4AII-NheI-NTD were used to amplify the full length mutant. The product was digested with NheI and XhoI and inserted into the same sites in pET28a.

For pET28a/eIF4AIII^IP/T^, two PCR products were produced using pET28a/eIF4AII as template and the primer pairs: (i) Primer D and Primer F and (ii) Primer E and Primer A (Table S1). Following gel purification, the fragments were used to extend off of each during 5 rounds of PCR amplification, after which the primers Primer A and Primer D were used to amplify the full length mutant. The product was digested with BamHI and inserted into the same site in pET28a to generate pET28a/eIF4AIII^IP^. The T mutation was introduced (Fig. S1) using pET28a/eIF4AIII^IP^ as template and the primer pairs: (i) Primer D and Primer C and (ii Primer B and Primer A (Table S1). Following gel purification, the fragments were used to extend off of each during 5 rounds of PCR amplification, after which the primers Primer A and Primer D were used to amplify the full length mutant. The product was digested with BamHI and inserted into the same site in pET28a. For pET28a/eIF4AIII^TLLQV^, two PCR products containing the TLLQV mutation were produced from pET28a/eIF4AIII: one with the primers Primer D and 4AIII(TLLQV)AS and the other with 4AIII(TLLQV)S and Primer A. Following gel purification, the fragments were used to extend off of each during 5 rounds of PCR amplification, after which the primers Primer A and Primer D were used to amplify the full length mutant. The product was digested with BamHI and inserted into the same site in pET28a.

### Purification of Hippuristanol

Hippuristanol was extracted from the gorgonian *I. hippuris* as previously described[20].

### Recombinant Protein Expression and Purification

Recombinant His_6_-eIF4AI and His_6_-eIF4AII were expressed in *E. Coli* BL21 (DE3) codon+. Bacteria were grown to an OD_600 _of 0.6 and induced with 1mM IPTG. Growth was continued an additional 3h at 37°C. Wild-type and mutant eIF4AI and eIF4AII proteins were resuspended in sonication buffer (20 mM Tris_7.5_, 10% glycerol, 0.1 mM EDTA, 200 mM KCl, 0.1% Triton X-100 and 3.4 mM β-mercaptoethanol). After sonication (9 pulses of 20 sec) and clarification by centrifugation (twice at 27,000×g for 30 min), the lysate was loaded on a Ni^++^-NTA agarose (Qiagen) column. The column was washed with 10 column volumes of wash 1 (20 mM Tris_7.5_, 10% glycerol, 0.1 mM EDTA, 800 mM KCl, 20 mM imidazole) and wash 2 (Wash 1 containing 300 mM KCl) buffer, and the His_6_-tagged proteins were eluted (Wash 1 buffer containing 300 mM KCl and 0.2 M imidazole), and dialyzed into A100 buffer (20 mM Tris_7.5_, 10% glycerol, 0.1 mM EDTA, 100 mM KCl, 2 mM DTT). The protein was loaded onto a Q-Sepharose Fast Flow (Amersham) column and eluted with a KCl gradient A100 to A500 (20 mM Tris_7.5_, 10% glycerol, 0.1 mM EDTA, 2 mM DTT ). The eluted protein was dialysed against 20 mM Tris_7.5_, 0.1 mM EDTA, and 10% glycerol.

Recombinant His_6_-eIF4AIII and mutants were purified through a Ni^++^-NTA agarose (Qiagen) column as previously described [33]. The eluent was diluted to a salt concentration of 100 mM and passed through a Q-Sepharose Fast Flow (Amersham) column and eIF4AIII collected in the flow through. His_6_-eIF4AIII was stored in 20 mM Tris_7.5_, 0.1 mM EDTA, 100 mM KCl, 10 % glycerol, and 2 mM DTT. His_6_-eIF4AIII^TLLQV^ was further purified on a heparin-sepharose column.

The cDNA encoding hDDX52 [NM007010.2] was obtained from Origene (Rockville, MD) and subcloned into the Not1 sites of pET28a. The purification procedure was as described above for eIF4AI using Ni^++^-NTA agarose and Q-Sepharose. Recombinant hDDX52 protein was stored in 20 mM Tris_7.5_, 10% glycerol, 0.1 mM EDTA, 2 mM DTT, 100mM KCl.

### ATPase, RNA binding, and Helicase Assays

ATPase assays were performed as described by Lorsch and Herschlag, using their “Condition B”[34]. Briefly, 0.1 µg or 1 µg protein (indicated in figure legend) was incubated with 2.5 µM poly (U) and 1 µM γ-^32^P-ATP (10 Ci/mmol) (0.01 µCi) at 25°C (except where specifically indicated) and time points taken at the indicated intervals by removing 2 µL aliquots and diluting into 2 µL of 25 mM EDTA. Inorganic phosphate and γ-^32^P-ATP were separated by TLC as described previously [34]. Results were quantitated using a Fujix BAS 2000 phosphoimager with a Fuji imaging screen. The fraction of P_i_ at t = 0 was typically 1–3% and subtracted as background.

For Tif1/2p, 0.5 µg Tif1/2p and 1 µg yeast eIF4G (aa542-883) was incubated with 12 nM 18S rRNA and 1 mM ATP at 25°C for 1h. The reaction was stopped with 50 mM EDTA and the formation of free phosphate determined as ammonium molybdate complex photometrically with malachite green (at A_630_) Calibration curves were prepared with increasing concentrations of Na_3_PO_4_ (5–100 µM) (BioAssays Systems).

Chemically cross-linking was performed with 0.5–1 µg recombinant protein and oxidized ^32^P-labelled CAT mRNA (∼18,000 cpm/µg) in the presence of 0.9 mM ATP with 10 µM hippuristanol or vehicle (DMSO) for 10 min at 30°C [20], [35], after which time sodium cyanoborohydride was added, and the incubation continued overnight at 4°C. The samples were then treated with RNAse A, separated on a 10% SDS-PAGE, and visualized by autoradiography.

RNA helicase assays were performed as previously described [7], [36]. Briefly, 0.4 µM recombinant His_6_-eIF4AI or His_6_-eIF4AII was incubated with 2 µM RNA-1/11 duplex in the presence of 1 mM ATP for 15 minutes at 35°C. Reactions were resolved on native 12% polyacrylamide gels, which were dried and exposed to X-Omat (Kodax) film at -70°C.

### Rescue of hippuristanol-induced translation inhibition

The plasmid pKS/FF/HCV/Ren was linearized with BamHI and transcribed with T3 RNA polymerase to generate FF/HCV/Ren mRNA [20]. *In vitro* translations were performed in rabbit reticulocyte lysates following the manufacturer’s instructions (Promega). Extracts were programmed with FF/HCV/Ren mRNA (8 µg/ml) and translations performed at a final concentration of 135 mM KCl. eIF4A rescue experiments were performed by the addition of 0.5 or 0.8 µg recombinant eIF4A (0.9–1.4 µM) to vehicle- or hippuristanol -treated extracts. Firefly luciferase activities were measured on a Berthold Lumat LB 9507 luminometer. Reactions performed in the presence of [^35^S]methionine were separated on a 10% SDS-polyacrylamide gel which was treated with EN^3^Hance, dried, and exposed to X-Omat (Kodax) film at −70°C. For *in vitro* translation reactions in yeast, wild type strain BY4741 was used to prepare an *in vitro* translation extract and assays were performed as previously reported [37].

Translations in *Xenopus* oocytes were performed essentially as previously described [38]. Briefly, collagenased *Xenopus* oocytes were sorted and incubated for 4 h at 16°C in 5 µM hippuristanol or 0.05% DMSO. Each oocyte was then injected with 50 nL of 0.94 mg/mL recombinant His_6_-eIF4AI or recombinant His_6_-eIF4AI^IG/T^ or buffer alone immediately followed by 10 nL of 0.02 µM in vitro transcribed FF/HCV/Ren mRNA. Oocytes were then incubated for 4h at room temperature in fresh compound dilutions. Three oocytes were homogenized in 150 µL Passive Lysis Buffer (Promega). The cell lysates were cleared by centrifugation at 14000×g for 5 min. Ten microliters of the lysate was read per sample using the Dual Luciferase Assay system (Promega). Values were normalized to *Renilla* activity and the percent rescue determined as average value of compound challenged samples divided by the average value of DMSO challenged samples.

#### Time resolved fluorescence resonance energy transfer (TR-FRET)

Recombinant His_6_-eIF4AI protein (20 nM ) and GST-eIF4GI_517-606 _(40 nM) or GST-eIF4GI_648–983 _(200 nM) were incubated with Eu-W1024 labeled anti-6xHis antibody (1 nM) [Perkin Elmer] and anti-GST IgG antibody conjugated to SureLight-Allophycocyanin (100 nM) [Perkin Elmer] in TR-FRET buffer (20 mM Hepes_7.4_, 10 mM KCl, 1 mM DTT, 0.015% Tween 20, 1 µg/ml IgG). Reactions were performed at room temperature for 3 hrs. FRET signal was monitored using an Analyst HT reader (LJL Biosystems) [39]. Data collection using the “Criterion Host v.2.0.1” software (LJL Biosystems) involved setting the Z height at 1 mm and utilizing 1 excitation filter (330/80) and 2 emission filters (620/7.5 and 665/7.5). A dichroic filter with a wavelength of 400 nm was used. For the measurement at 620 nm we employed 100 readings per well, with 10 ms between reading, integration time of 1000 µs, a delay time of 200 µs and 1000 µs integration time for the fluorescence emission recording. The parameters for the measurement at 665 nm were the same as for 620 nm, except for an integration time of 150 µs and a delay time of 50 µs. Due to the time delay, only the longer-lived FRET signal is detected, eliminating short-lived background fluorescence. The 665/620 ratio was calculated and normalized to the negative control reaction (containing His_6_-eIF4E_W73A_ which does not interact with eIF4G or 4E-BP) to yield the S/B ratio. The 665 nm emissions are due to APC FRET and the 620 nm emissions are due to Eu-W1024 fluorescence.

## Supporting Information

Table S1 (0.05 MB DOC)

Figure S1 Amino acid alignment of the hippuristanol binding site among murine eIF4AI, murine eIF4AII, and human eIF4AIII alleles used in this study. Direct protein-hippuristanol NOEs are highlighted in yellow, whereas those within 5{Angstrom} are in grey. Introduced amino acid changes are highlighted in red. The position of the first and last amino acid of the motif is indicated. Amino acids corresponding to the hippuristanol binding site in *S. cerevisiae* Ded1p is also shown.
(0.03 MB DOC)

Figure S2 Amino acid alignment of the hippuristanol binding site among DDX family members. Alignments shown are for members of the murine (A) or human (B) DEAD box family members. The Entrez Protein IDs are provided in parenthesis for each member. Direct protein-hippuristanol NOEs are highlighted in yellow, whereas those within 5{Angstrom} are in grey. The position of the first and last amino acid of the motif in the protein sequence is indicated.
(0.07 MB DOC)

Figure S3 RNA-dependent ATPase activity of eIF4AI^IG/T^, eIF4AI^IG^, and eIF4AI^T^ mutant alleles. ATP hydrolysis was monitored using 1 µg recombinant protein. Each value represents the average of two measurements with the error of the mean presented. [Note in this assay, the protein preparation was different and not as active as the preparation used in Fig. 3A.]
(10.00 MB TIF)

Figure S4 Characterization of eIF4AI and eIF4AII hippuristanol-resistant mutants. (A) Crosslinking of recombinant proteins to RNA in the presence of hippuristanol. ^32^P-labelled CAT RNA was cross-linked to 0.5–1 µg of the indicated recombinant protein in the presence or absence of hippuristanol, separated by SDS-PAGE, and visualized by autoradiography. (B) The helicase activities of the eIF4AI^IG/T^ and eIF4AII^IP/T^ mutants are resistant to hippuristanol. Helicase assays were performed with recombinant protein (0.4 µM) and duplexed RNA as described in the Materials and Methods. Reactions were resolved on a native 12% acrylamide gel, which was dried, and exposed to BioMax XAR film (Kodak) film at −70°C. The position of migration of duplexed (ds) and single-stranded (ss) RNA are denoted to the right.
(5.78 MB TIF)

Figure S5 Characterization of eIF4A mutants. (A) RNA-dependent ATPase activity of eIF4AI^Hel/IG/T^ and eIF4AI^Quad/IG/T^ mutants. ATP hydrolysis was monitored using 1 µg recombinant protein. Each value represents the average of two measurements with the error of the mean presented. In this experiment, the protein preparations were different and not as active as the preparations used in Fig. 3A. (B) The helicase activity of eIF4AI^Hel/IG/T^ is impaired. Recombinant protein (0.4 µM) was incubated with duplexed RNA as described in Materials and Methods. Reactions were resolved on a native 12% acrylamide gel and visualized by autoradiography. The migration of duplexed and ssRNA are determined by the incubation of duplexed RNA alone at 35°C (lane 1) or boiling for 5 minutes (lane 2), respectively. (C) Crosslinking of eIF4AI^Quad/IG/T^ and eIF4AI^Hel/IG/T^ to RNA in the presence of hippuristanol. ^32^P-labelled CAT RNA was cross-linked to 1 µg of the indicated recombinant protein in the presence or absence of hippuristanol, separated by SDS-PAGE, and visualized by autoradiography. (D) Helicase activity of eIF4AI^Quad/IG/T^ is not impaired and resistant to hippuristanol. Helicase assays were performed with recombinant protein (0.4 µM) and duplexed RNA as described in the Materials and Methods. Reactions were resolved on a native 12% acrylamide gel, which was dried, and exposed to BioMax XAR film (Kodak) film at −70°C. The position of migration of duplexed (ds) and single-stranded (ss) RNA are denoted to the right.
(4.08 MB TIF)

Figure S6 The interaction of eIF4AI^Quad/IG/T^ with eIF4GI is impaired. (A) Schematic representation of the various functional domains of eIF4GI. Protein and RNA binding sites on eIF4GI are indicated. The numbers below eIF4GI refer to the amino acid location of each binding site. A schematic of the recombinant eIF4GI fragments utilized and the regions they span are shown in grey boxes. (B) TR-FRET analysis of the interaction between eIF4AI, eIF4AI^IG/T^, eIF4AI^Quad/IG/T^ with eIF4GI fragments. GST-eIF4GI fragments were incubated with recombinant His_6_-eIF4AI protein, as well as with Eu-W1024 labeled anti-6xHis antibody and anti-GST IgG antibody conjugated to SureLight-Allophycocyanin. The FRET signal (expressed as the signal to background ratio (S/B)) was monitored on an Analyst reader (LJL Biosystems) and represents the average of 4 experiments with the standard error of the mean shown. The signal obtained with eIF4AI and eIF4G_517-606_ was equivalent to the background signal (S/B = 1).
(4.39 MB TIF)
