# Supplementary material for: Selective Pharmacological Targeting of a DEAD Box RNA Helicase
Source: PLoS One. 2008 Feb 13;3(2):e1583. doi: 10.1371/journal.pone.0001583 (PMC2216682; doi:10.1371/journal.pone.0001583)
Supplement: Table S1 — (0.05 MB DOC) [file pone.0001583.s001.doc]

**Table S1**. Table of primer sequences used in this study.

| Primer name | Sequence |
| --- | --- |
| **eIF4AI** |  |
| IG Forward | 5′-gcc aga ggc att gat att ggg cag gtc tcc tta gtc atc aac tat gac-3′ |
| IG Reverse | 5′-gac taa gga gac ctg ccc aat atc aat gcc tct ggc caa cag g-3′ |
| IP Forward | 5′-gcc aga ggc att gat att ccg cag gtc tcc tta gtc atc aac tat gac-3′ |
| IP Reverse | 5′-gac taa gga gac ctg cgg aat atc aat gcc tct ggc caa cag g-3′ |
| T Forward | 5′-cag aga atc ggt cga act ggt cgg ttt ggt cgt aag ggt g-3′ |
| T Reverse | 5′-acg acc aaa ccg acc agt tcg acc gat tct gtg gat gta g-3′ |
| DraIII Forward | 5′-gga agc ttg aca cat tgt gtg act tg-3′ |
| BamHI Reverse | 5′-ggc ttt gtt agc agc cgg atc c-3′ |
| D265R/E268Kforward | 5′-gac aca ttg tgt cgc ttg tat aag acg ctg acc atc-3′ |
| D265R/E268Kreverse | 5′-gat ggt cag cgt ctt ata caa gcg aca caa tgt gtc-3′ |
| 4A(1220-1238)(AS) | 5′-cgg gga tcc agc ccc tca aat gag gtc ag-3′ |
| pET15b Oligo | 5′-cgg aag ctt agc ggc ctg gtg ccg cg-3′ |
| D296A/T298Kforward | 5′-atg cat gcc cga gct ttc aag gtt tgt gcc atg-3′ |
| D296A/T298Kreverse | 5′-cat ggc aga aac ctt gaa agc tcg ggc atg cat-3′ |
| AIGforward | 5′-gcc aga ggc att gct att ggg cag gtc tcc taa-3′ |
| AIGreverse | 5′-taa gga gac ctg ccc aat agc aat gcc tct ggc-3′ |
|  |  |
| **eIF4AII** |  |
| 4AII-T(S) | 5′-ata ttc aca gaa ttg gca gaa cag gtc gat ttg gga gga aag-3′ |
| 4AII-T(AS) | 5′-ctt tcc tcc caa atc gac ctg ttc tgc caa ttc tct gaa tat-3′ |
| 4AII-IP(S) | 5′-ctt gtt ggc ccg tgg gat tga cat tcc gca agt gtc ctt ggt tat aaa c-3′ |
| 4AII-IP(AS) | 5′-gtt tat aac caa gga cac ttg cgg aat gtc aat ccc acg ggc caa caa g-3′ |
| 4AII-NheI-NTD | 5′-cgg gct agc atg tct ggt ggc tcc gcg-3′ |
| 4AII(1278-1258) | 5′-ggc ctc gag caa aac tat ctc atc cca ggg-3′ |
|  |  |
| **eIF4AIII** |  |
| Primer A | 5′-ttg ttg gga tcc tca gat aag atc agc aac gtt cat c-3′ |
| Primer B | 5′-ggg aga act ggt cga tac ggc cgg aag-3′ |
| Primer C | 5′-gta tcg acc agt tct ccc aat tct gtg tat gta-3′ |
| Primer D | 5′-ggt cgc gga tcc atg g-3′ |
| Primer E | 5′-ttg gat atc cct cag gtg tcc ctc-3′ |
| Primer F | 5′-aca cct gag gga tat cca acc ccc tgg c-3′ |
| 4AIII(TLLQV)AS | 5′-gac atc gat ccc cct ggc cag gag atc tgt agt aat aag cac tcg gct ggc-3′ |
| 4AIII(TLLQV)S | 5′-cca ggg gga tcg atg tcc agc agg tgt ccc tcg tca tta act atg atc tcc cta ata ac-3′ |
