# Supplementary material for: Selective Pharmacological Targeting of a DEAD Box RNA Helicase
Source: PLoS One. 2008 Feb 13;3(2):e1583. doi: 10.1371/journal.pone.0001583 (PMC2216682; doi:10.1371/journal.pone.0001583)
Supplement: Figure S1 — Amino acid alignment of the hippuristanol binding site among murine eIF4AI, murine eIF4AII, and human eIF4AIII alleles used in this study. Direct protein-hippuristanol NOEs are highlighted in yellow, whereas those within 5{Angstrom} are in grey. Introduced amino acid changes are highlighted in red. The position of the first and last amino acid of the motif is indicated. Amino acids corresponding to the hippuristanol binding site in S. cerevisiae Ded1p is also shown. (0.03 MB DOC) [file pone.0001583.s002.doc]

**V VI**

eIF4AI 328-TTDLL**ARGID**VQQVSLVIN-(11)-**HRIGRGGRFG**RKGVAINM-375

eIF4AI^IG^ 328-TTDLLARGIDIGQVSLVIN-(11)-HRIGRGGRFGRKGVAINM-375

eIF4AI^T^ 328-TTDLLARGIDVQQVSLVIN-(11)-HRIGRTGRFGRKGVAINM-375

eIF4AI^IG/T^ 328-TTDLLARGIDIGQVSLVIN-(11)-HRIGRTGRFGRKGVAINM-375

eIF4AI^IP/T^ 328-TTDLLARGIDIPQVSLVIN-(11)-HRIGRTGRFGRKGVAINM-375

eIF4AI^Hel/IG/T^ 328-TTDLLARGIAIGQVSLVIN-(11)-HRIGRTGRFGRKGVAINM-375

eIF4AI^Quad/IG/T^ 328-TTDLLARGIDIGQVSLVIN-(11)-HRIGRTGRFGRKGVAINM-375

Tif1/2p 316-STDLLARGIDVQQVSLVIN-(11)-HRIGRGGRFGRKGVAINF-363

eIF4AII 329-TTDLLARGIDVQQVSLVIN-(11)-HRIGRGGRFGRKGVAINF-376

eIF4AII^IP/T^ 329-TTDLLARGIDIPQVSLVIN-(11)-HRIGRTGRFGRKGVAINF-376

eIF4AIII 333–stdvwargldvpqvsliin------hrigrsgrygrkgvainf-380

eIF4AIII^IP/T^  333–stdvwargldIpqvsliin------hrigrTgrygrkgvainf-380

eIF4AIII^TTLQV^ 333–TTDLLARGIDVQQVSLVIN------hrigrsgrygrkgvainf-380

Ded1p 455-ATAVAARGLDIPNVTHVIN------HRIGRTGRAGNTGLATAF-502
