# Supplementary material for: Selective Pharmacological Targeting of a DEAD Box RNA Helicase
Source: PLoS One. 2008 Feb 13;3(2):e1583. doi: 10.1371/journal.pone.0001583 (PMC2216682; doi:10.1371/journal.pone.0001583)
Supplement: Figure S2 — Amino acid alignment of the hippuristanol binding site among DDX family members. Alignments shown are for members of the murine (A) or human (B) DEAD box family members. The Entrez Protein IDs are provided in parenthesis for each member. Direct protein-hippuristanol NOEs are highlighted in yellow, whereas those within 5{Angstrom} are in grey. The position of the first and last amino acid of the motif in the protein sequence is indicated. (0.07 MB DOC) [file pone.0001583.s003.doc]

**A. Murine DDX Family Members**

**V VI**

DDX2A (NP 659207)[eIF4AI] 328-TTDLL**ARGID**VQQVSLVIN-(11)-**HRIGRGGRFG**RKGVAINM-375

DDX2B (NP 038534)[eIF4AII] 329-TTDLLARGIDVQQVSLVIN-(11)-HRIGRGGRFGRKGVAINF-376

DDX48 (NP_619610)[eIF4AIII] 333-stdvwargldvpqvsliin------hrigrsgrygrkgvainf-380

DDX51 (Q6P9R1) 534-STDATARGIDVQGVELVIN------HRVGRTARAGKTGQAFTL-581

DDX25 (Q9QY15) 395-TTNVCARGIDVKQVTIVVN------HRIGRTGRFGKKGLAFNM-448

DDX46 (Q569Z5) 675-ATSVAARGLDVKHLILVVN------HRAGRTGRAGNKGYAYTF-722

DD19A (Q61655) 388-TTNVCARGIDVEQVSVVIN------HRIGRTGRFGKRGLAVNM-441

DDX52 (Q8K301) 469-CTALLARGIDFKGVNLVIN------HRIGRTGRAGNRGKAVTF-516

DDX23*(XP619355) 719-atdvagrgidiqdvsmvvn------hrigrtgragksgvaitf-766

DDX42 (Q3TAN3) 554-ATDVAARGLDIPSIKTVIN------HRIGRTGRAGEKGVAYTL-601

DDX17 (Q501J6) 395-ATDVASRGLDVEDVKFVIN------HRIGRTARSTNKGTAYTF-442

DDX20 (Q9JJY4) 366-STDLTSRGIDAEKVNLVVN------HRIGRAGRFGTLGLTVTY-413

DDX21 (Q9JIK5) 565-ATNVAARGLDIPEVDLVVQ------HRSGRTGRAGRTGVCICF-612

DDX6 (P54823) 390-CTDLFTRGIDIQAVNVVIN------HRIGRSGRFGHLGLAINL-437

DDX1 (Q91VR5) 571-CTDVAARGIDIHGVPYVIN------HRIGRVGRAERMGLAISL-618

DDX56 (Q9D0R4) 344-ATDGVARGIDFHHVSAVLN------HRAGRTARANNPGIVLTF-388

DDX5 (Q4R6M5) 397-ATDVASRGLDVEDVKFVIN------HRIGRTARSTKTGTAYTF-444

DDX43^#^ (XP001060057) 541-atdlasrgldvhdithvyn------hrvgrtgragrtgmsitl-588

DDX59 (NP080776) 501-stgvlgrgldlvnvklvvn------hqvgrvgrlgqngtaitf-548

DDX39 (Q8VDW0) 342-ATNLFGRGMDIERVNIVFN------HRVARAGRFGTKGLAVTF-389

DDX55 (Q6ZPL9) 324-CTDVMARGIDIPEVNWVLQ------HRCGRTARIGHGGSALVF-371

DDX50 (NP444413) 441-atnvaargldipevdlviq------hrsgrtgragrtgicvcf-488

DDX54 (Q8K4L0) 393-VTDLAARGLDIPLLDNVIN------HRVGRVARAGRSGTAYSL-440

DDX49 (Q4FZF3) 304-ATDVASRGLDIPTVQVVIN------HRVGRTARAGRQGQAITL-351

DDX27 (Q921N6) 485-ATDVAARGLDIEGVKTVIN------HRVGRTARAGRAGRSVSL-532

DDX47 (Q9H0S4) 319-ATDVASRGLDIPHVDVVVN------HRVGRTARAGRSGKAITF-366

DDX4 (Q61496) 569-ATSVAARGLDIENVQHVIN------HRIGRTGRCGNTGRAISF-616

DDX3X (Q62167) 497-ATAVAARGLDISNVKHVIN------HRIGRTGRVGNLGLATSF-544

BAT1 (AAD30177) 343-atnlfgrgmdiervniafn------hrvaragrfgtkglaitf-390

DDX3Y (Q62095) 496-ATAVAARGLDISNVKHVIN------HRIGRTGRVGNLGLATSF-543

DDX10 (Q80Y44) 371-ATDIAARGLDFPAVNWVLQ------HRAGRTARYKEDGEALLI-418

DDX41 (Q91VN6) 488-ATDVASKGLDFPAIQHVIN------HRIGRTGRSGNTGIATTF-535

DDX24 (Q9ESV0) 643-ATDVAARGLDIPKVQHVIH------HRSGRTARAASEGLSLML-690

DDX28 (Q9CWT6) 453-CTDIASRGLDSVHVEVVIN------HRAGRVGRVGSEVPGSVI-500

DDX31 (AAH66017) 448-CTDVASRGLDLPQVTWIVQ------HRIGRTARIGCHGSSLLI-495

DDX18 (Q8K363) 471-CTDVAARGLDIPEVDWIVQ------HRVGRTARGLNGRGHALL-518

*Predicted by computational analysis

#Predicted by computational analysis of Rattus norvegicus sequence

**B. Human DDX Family Members**

**V VI**

DDX2A (NP 001407) [eIF4AI] 328-TTDLL**ARGID**VQQVSLVIN-(11)-**HRIGRGGRFG**RKGVAINM-375

DDX2B (NP 001958) [eIF4AII] 329-TTDLLARGIDVQQVSLVIN-(11)-HRIGRGGRFGRKGVAINF-376

DDX48 (NP 055555)[eIF4AIII] 333–stdvwargldvpqvsliin------hrigrsgrygrkgvainf-380

DDX52 (Q9Y2R4) 468-CTALLARGIDFKGVNLVIN------HRIGRTGRAGNKGKAITF-515

DDX25 (NP037396) 394-TTNVCARGIDVKQVTIVVN------HRIGRTGRFGKKGLAFNM-447

DDX51 (Q8N8A6) 561-STDATARGIDVQGVELVVN------HRVGRTARAGKTGQAFTL-608

DDX46 (Q7L014) 675-ATSVAARGLDVKHLILVVN------HRAGRTGRAGNKGYAYTF-722

DDX19 (Q9NUU7) 388-TTNVCARGIDVEQVSVVIN------HRIGRTGRFGKRGLAVNM-441

DDX23 (Q9BUQ8) 720-ATDVAGRGIDIQDVSMVVN------HRIGRTGRAGKSGVAITF-767

DDX43 (Q9NXZ2) 543-ATDLASRGLDVHDVTHVYN------HRIGRTGRAGRTGVSITT-590

DDX42 (Q68G51) 535-ATDVAARGLDIPSIKTVIN------HRIGRTGRAGEKGVAYTL-582

DDX17 (Q92841) 395-ATDVASRGLDVEDVKFVIN------HRIGRTARSTNKGTAYTF-442

DDX21 (Q9NR30) 493-ATNVAARGLDIPEVDLVIQ------HRSGRTGRAGRTGVCICF-540

DDX20 (Q9UHI6) 365-STDLTSRGIDAEKVNLVVN------HRIGRAGRFGTLGLTVTY-412

DDX6 (P26196) 390-CTDLFTRGIDIQAVNVVIN------HRIGRSGRFGHLGLAINL-437

DDX1 (Q92499) 571-ctdvaargidihgvpyvin------hrigrvgraermglaisl-618

DDX5 (P17844) 397-ATDVASRGLDVEDVKFVIN------HRIGRTARSTKTGTAYTF-444

DDX39 (O00148) 342-ATNLFGRGMDIERVNIVFN------HRVARAGRFGTKGLAITF-389

DDX59 (EAW91319) 501-stgvlgrgldlisvrlvvn------hqigrvgrlgqngtaitf-548

DDX55 (Q8NHQ9) 324-CTDVMARGIDIPEVNWVLQ------HRCGRTARIGHGGSALVF-371

DDX50 (AAH00210) 174-atnvaargldipevdlviq------hrsgrtgragrtgicicf-221

DDX56 (Q9NY93) 341-PEAGVARGIDFHHVSAVLN------HRAGRTARANNPGIVLTF-388

DDX7 (Q15320) 287-VTDVAARGLDIEDLPLVVN------HRAGRTGRMGKPGRVLNL-334

DDX54 (Q8TDD1) 394-VTDLAARGLDIPLLDNVIN------HRVGRVARAGRSGTAYSL-441

DDX49 (Q9Y6V7) 304-ATDVASRGLDIPTVQVVIN------HRVGRTARAGRQGQAITL-351

DDX27 (Q96GQ7) 519-ATDVAARGLDIEGVKTVIN------HRVGRTARAGRAGRSVSL-566

DDX47 (Q9H0S4) 319-ATDVASRGLDIPHVDVVVN------HRVGRTARAGRSGKAITF-366

DDX4 (Q9NQI0) 596-ATSVAARGLDIENVQHVIN------HRIGRTGRCGNTGRAISF-643

DDX3X (O00571) 497-ATAVAARGLDISNVKHVIN------HRIGRTGRVGNLGLATSF-544

DDX3Y (O15523) 495-ATAVAARGLDISNVRHVIN------HRIGRTGRVGNLGLATSF-542

BAT1 (AAX42258) 343-atnlfgrgmdiervniafn------hrvaragrfgtkglaitf-390

DDX53 (Q86TM3) 523-TTDIVSRGLDLNDVTHVYN------HRVGYIGRTGKTGTSVTL-570

DDX24 (Q9GZR7) 643-ATDVAARGLDIPKVQHVIH------HRSGRTARATNEGLSLML-690

DDX10 (Q13206) 371-ATDIAARGLDFPAVNWVLQ------HRAGRTARYKEDGEALLI-418

DDX28 (Q9NUL7) 453-CTDIASRGLDSTGVELVVN------HRAGRVGRVGSEVPGTVI-500

DDX41 (Q9UJV9) 488-ATDVASKGLDFPAIQHVIN------HRIGRTGRSGNTGIATTF-535

DDX31 (Q9H8H2) 584-CTDVAARGLDLPQVTWIVQ------HRIGRTARIGCHGSSLLI-631

DDX18 (Q9NVP1) 481-CTDVAARGLDIPEVDWIVQ------HRVGRTARGLNGRGHALL-528
