# Supplementary figures and images for: Selective Pharmacological Targeting of a DEAD Box RNA Helicase
Source: PLoS One. 2008 Feb 13;3(2):e1583. doi: 10.1371/journal.pone.0001583 (PMC2216682; doi:10.1371/journal.pone.0001583)

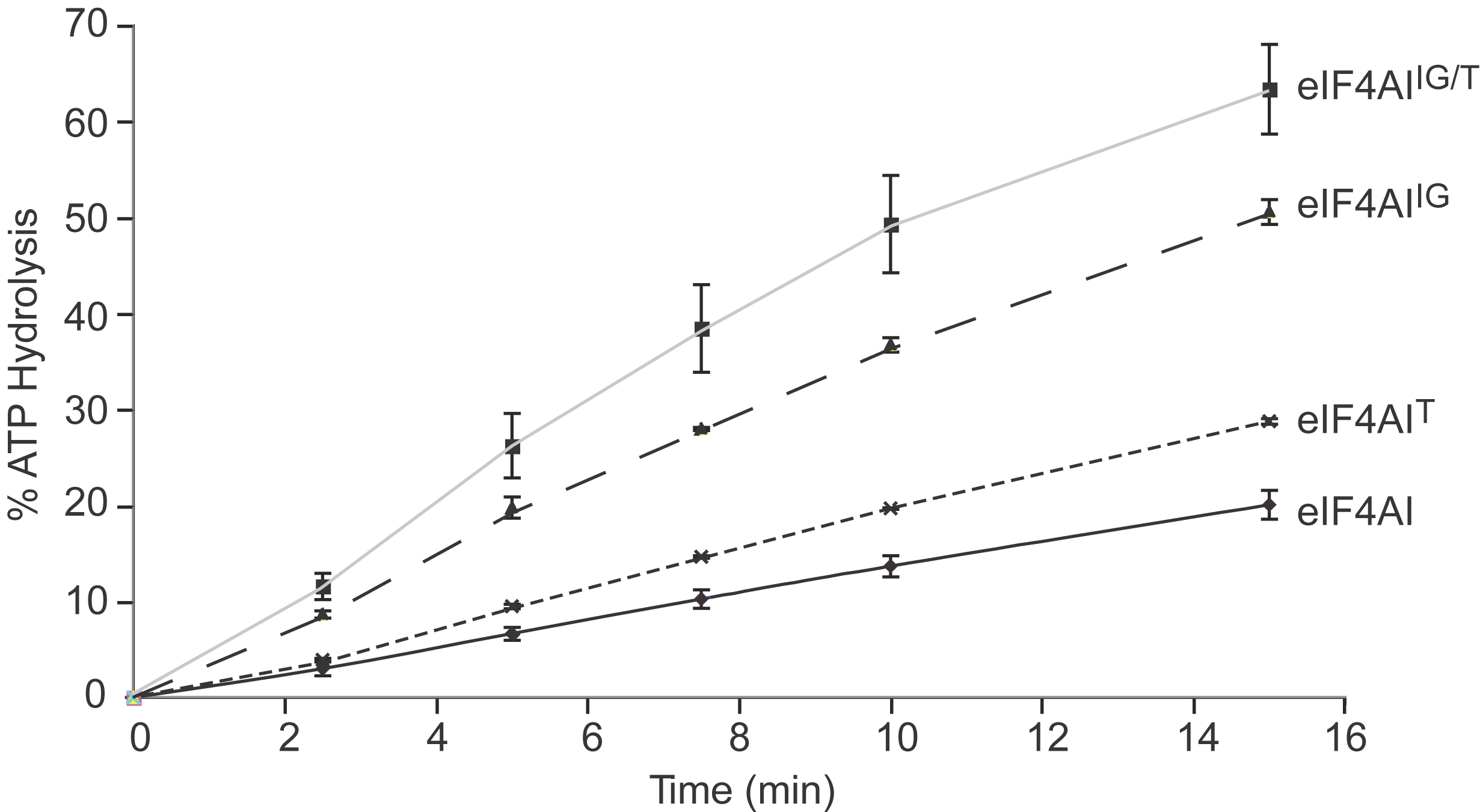

Supplement: Figure S3 — RNA-dependent ATPase activity of eIF4AIIG/T, eIF4AIIG, and eIF4AIT mutant alleles. ATP hydrolysis was monitored using 1 µg recombinant protein. Each value represents the average of two measurements with the error of the mean presented. [Note in this assay, the protein preparation was different and not as active as the preparation used in Fig. 3A.] (10.00 MB TIF) [file pone.0001583.s004.tif]

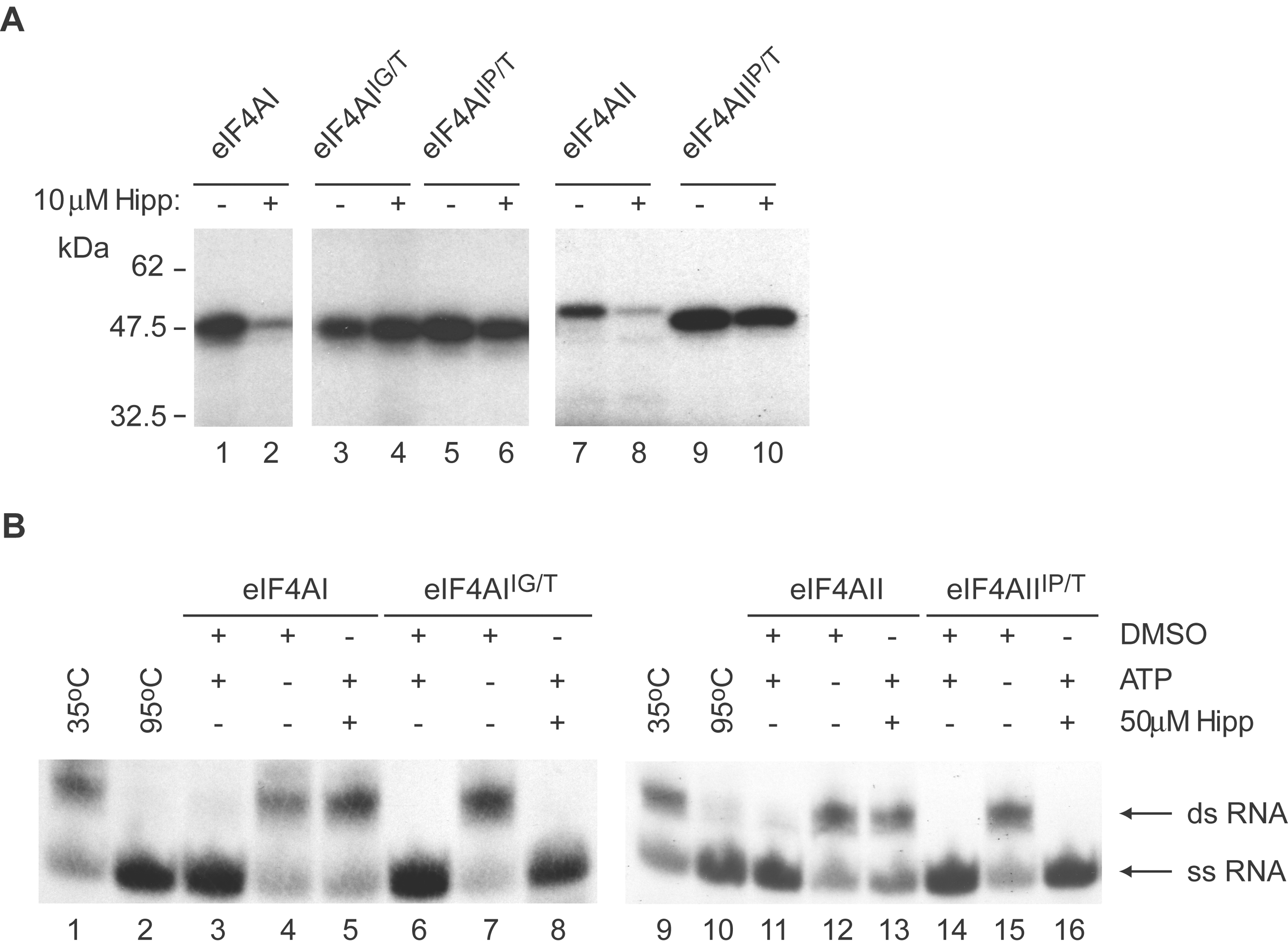

Supplement: Figure S4 — Characterization of eIF4AI and eIF4AII hippuristanol-resistant mutants. (A) Crosslinking of recombinant proteins to RNA in the presence of hippuristanol. 32P-labelled CAT RNA was cross-linked to 0.5–1 µg of the indicated recombinant protein in the presence or absence of hippuristanol, separated by SDS-PAGE, and visualized by autoradiography. (B) The helicase activities of the eIF4AIIG/T and eIF4AIIIP/T mutants are resistant to hippuristanol. Helicase assays were performed with recombinant protein (0.4 µM) and duplexed RNA as described in the Materials and Methods. Reactions were resolved on a native 12% acrylamide gel, which was dried, and exposed to BioMax XAR film (Kodak) film at −70°C. The position of migration of duplexed (ds) and single-stranded (ss) RNA are denoted to the right. (5.78 MB TIF) [file pone.0001583.s005.tif]

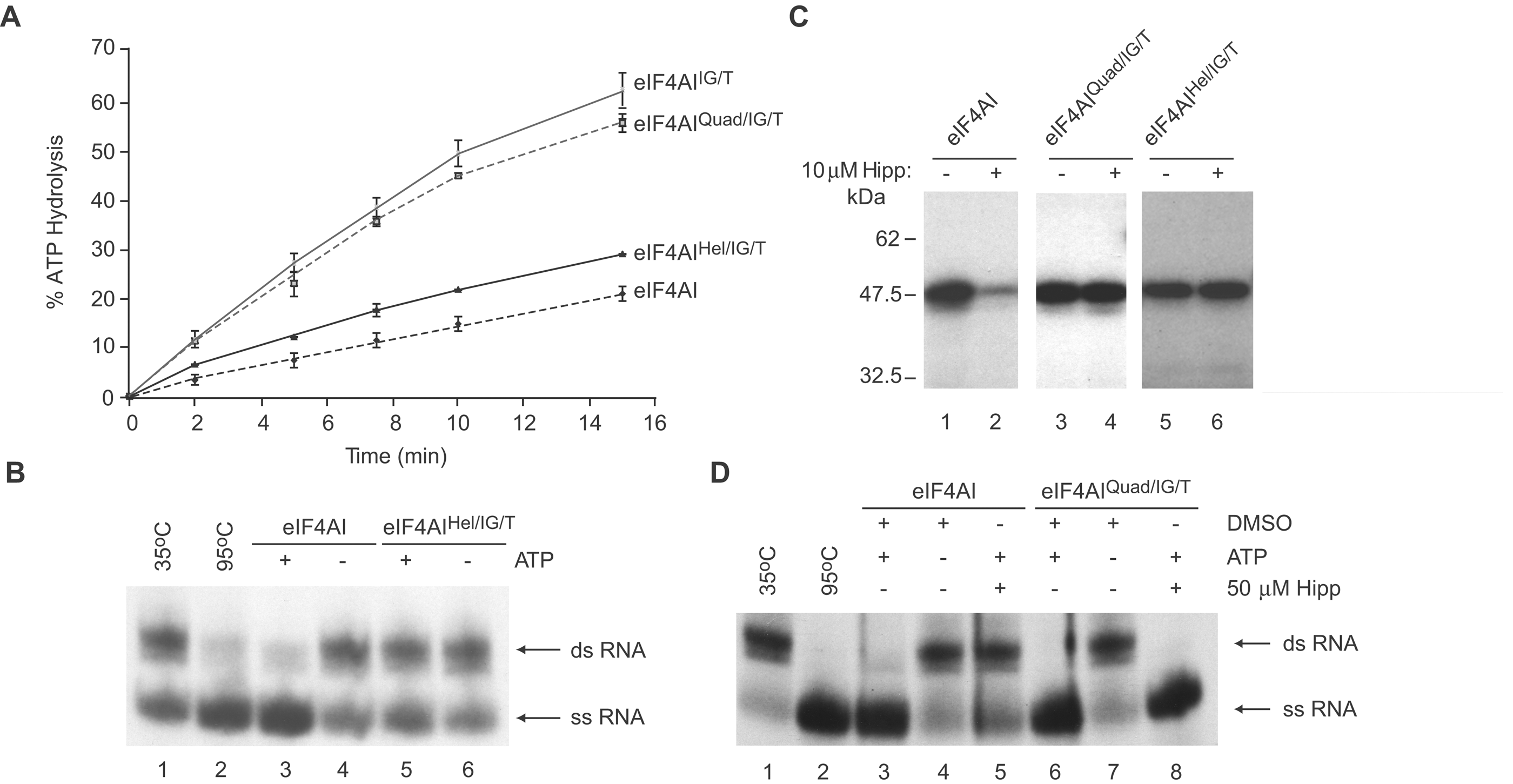

Supplement: Figure S5 — Characterization of eIF4A mutants. (A) RNA-dependent ATPase activity of eIF4AIHel/IG/T and eIF4AIQuad/IG/T mutants. ATP hydrolysis was monitored using 1 µg recombinant protein. Each value represents the average of two measurements with the error of the mean presented. In this experiment, the protein preparations were different and not as active as the preparations used in Fig. 3A. (B) The helicase activity of eIF4AIHel/IG/T is impaired. Recombinant protein (0.4 µM) was incubated with duplexed RNA as described in Materials and Methods. Reactions were resolved on a native 12% acrylamide gel and visualized by autoradiography. The migration of duplexed and ssRNA are determined by the incubation of duplexed RNA alone at 35°C (lane 1) or boiling for 5 minutes (lane 2), respectively. (C) Crosslinking of eIF4AIQuad/IG/T and eIF4AIHel/IG/T to RNA in the presence of hippuristanol. 32P-labelled CAT RNA was cross-linked to 1 µg of the indicated recombinant protein in the presence or absence of hippuristanol, separated by SDS-PAGE, and visualized by autoradiography. (D) Helicase activity of eIF4AIQuad/IG/T is not impaired and resistant to hippuristanol. Helicase assays were performed with recombinant protein (0.4 µM) and duplexed RNA as described in the Materials and Methods. Reactions were resolved on a native 12% acrylamide gel, which was dried, and exposed to BioMax XAR film (Kodak) film at −70°C. The position of migration of duplexed (ds) and single-stranded (ss) RNA are denoted to the right. (4.08 MB TIF) [file pone.0001583.s006.tif]

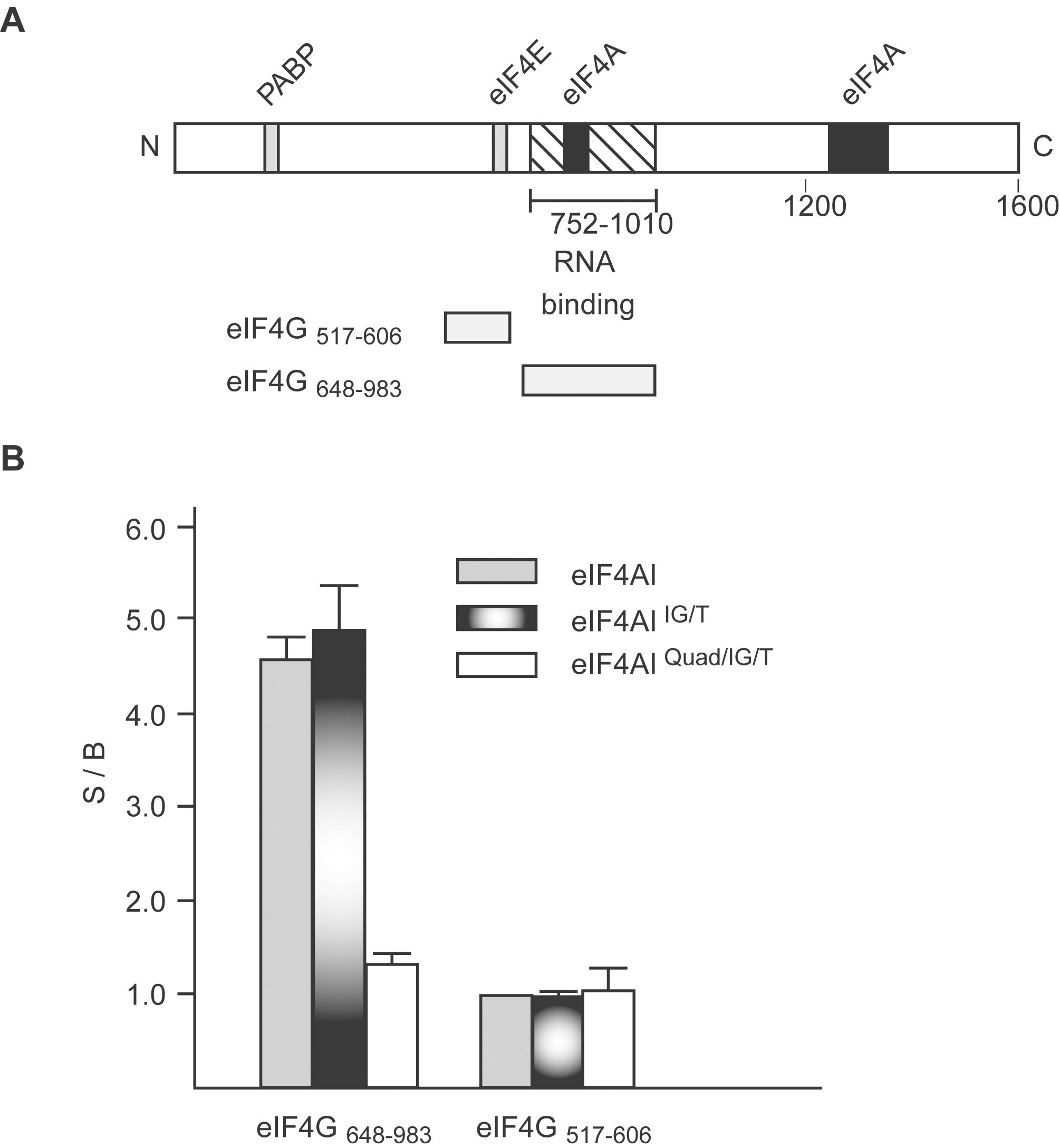

Supplement: Figure S6 — The interaction of eIF4AIQuad/IG/T with eIF4GI is impaired. (A) Schematic representation of the various functional domains of eIF4GI. Protein and RNA binding sites on eIF4GI are indicated. The numbers below eIF4GI refer to the amino acid location of each binding site. A schematic of the recombinant eIF4GI fragments utilized and the regions they span are shown in grey boxes. (B) TR-FRET analysis of the interaction between eIF4AI, eIF4AIIG/T, eIF4AIQuad/IG/T with eIF4GI fragments. GST-eIF4GI fragments were incubated with recombinant His6-eIF4AI protein, as well as with Eu-W1024 labeled anti-6xHis antibody and anti-GST IgG antibody conjugated to SureLight-Allophycocyanin. The FRET signal (expressed as the signal to background ratio (S/B)) was monitored on an Analyst reader (LJL Biosystems) and represents the average of 4 experiments with the standard error of the mean shown. The signal obtained with eIF4AI and eIF4G517-606 was equivalent to the background signal (S/B = 1). (4.39 MB TIF) [file pone.0001583.s007.tif]
